# The yeast mitogen-activated protein kinase Slt2 is involved in the cellular response to genotoxic stress

**DOI:** 10.1186/1747-1028-7-1

**Published:** 2012-02-01

**Authors:** María Soriano-Carot, M Carmen Bañó, J Carlos Igual

**Affiliations:** 1 Departament de Bioquímica i Biologia Molecular. Universitat de València. 46100 Burjassot (Valencia), Spain

**Keywords:** Slt2, genotoxic stress, DNA damage, checkpoint, *Saccharomyces cerevisiae*

## Abstract

**Background:**

The maintenance of genomic integrity is essential for cell viability. Complex signalling pathways (DNA integrity checkpoints) mediate the response to genotoxic stresses. Identifying new functions involved in the cellular response to DNA-damage is crucial. The *Saccharomyces cerevisiae SLT2 *gene encodes a member of the mitogen-activated protein kinase (MAPK) cascade whose main function is the maintenance of the cell wall integrity. However, different observations suggest that *SLT2 *may also have a role related to DNA metabolism.

**Results:**

This work consisted in a comprehensive study to connect the Slt2 protein to genome integrity maintenance in response to genotoxic stresses. The *slt2 *mutant strain was hypersensitive to a variety of genotoxic treatments, including incubation with hydroxyurea (HU), methylmetanosulfonate (MMS), phleomycin or UV irradiation. Furthermore, Slt2 was activated by all these treatments, which suggests that Slt2 plays a central role in the cellular response to genotoxic stresses. Activation of Slt2 was not dependent on the DNA integrity checkpoint. For MMS and UV, Slt2 activation required progression through the cell cycle. In contrast, HU also activated Slt2 in nocodazol-arrested cells, which suggests that Slt2 may respond to dNTP pools alterations. However, neither the protein level of the distinct ribonucleotide reductase subunits nor the dNTP pools were affected in a *slt2 *mutant strain. An analysis of the checkpoint function revealed that Slt2 was not required for either cell cycle arrest or the activation of the Rad53 checkpoint kinase in response to DNA damage. However, *slt2 *mutant cells showed an elongated bud and partially impaired Swe1 degradation after replicative stress, indicating that Slt2 could contribute, in parallel with Rad53, to bud morphogenesis control after genotoxic stresses.

**Conclusions:**

Slt2 is activated by several genotoxic treatments and is required to properly cope with DNA damage. Slt2 function is important for bud morphogenesis and optimal Swe1 degradation under replicative stress. The MAPK Slt2 appears as a new player in the cellular response to genotoxic stresses.

## Background

Genome stability and integrity maintenance are fundamental tasks in the cellular function. The DNA in each cell is under constant attack: genomic transactions, spontaneous chemical changes in DNA constituents, replication defects, and endogenous and exogenous agents, inflict damage to DNA. An efficient response to DNA damage is crucial to maintain cellular viability and to prevent diseases like cancer. Eukaryotic cells have developed surveillance mechanisms to respond to genotoxic stresses. These are the DNA damage and DNA replication checkpoints (referred to as DNA integrity checkpoints), a complex signaling network that coordinates cell cycle progression with DNA repair in response to DNA damage or defects in DNA replication to avoid genomic instability [1].

Checkpoint machinery is highly conserved in eukaryotes. The major regulators of the DNA-damage response are the PI3K-related protein kinases ATM (ataxia-telangiectasia mutated) and ATR (ATM and RAD3-related) kinases, Tel1 and Mec1, respectively in *S. cerevisiae *[2-5]. Tel1 and Mec1 have overlapping yet distinct functions in maintaining yeast genome integrity. Tel1 is specific in signaling double-strand breaks (DSBs). In contrast, Mec1 plays a more general role by functioning in the response to different types of damage, including DSBs, base adducts or crosslinks, and functions during the S phase to regulate the firing of replication origins. Early in the response, Mec1 and Tel1 are recruited to the sites of DNA damage together with accessory proteins that provide platforms on which damage response components are assembled. A final consequence is that Mec1 and Tel1 phosphorylate and activate the checkpoint effector kinases Chk1 and Rad53 [6]. Rad53 mediates most of the response in budding yeast cells. Once phosphorylated, Rad53 is released from chromatin to act on critical targets that promote cell cycle arrest. Additionally, Rad53 targets factors to induce the expression of DNA repair genes, stimulates deoxyribonucleotide triphosphate (dNTP) production, suppresses the replication origins firing and stabilizes replication forks.

In most eukaryotic cells, cell cycle progression is blocked in response to DNA damage or replication stress mainly by stimulating inhibitory phosphorylation of cyclin-dependent kinases (Cdc28 in *S. cerevisiae*). This inhibition is controlled by the balance between the inhibitory Wee1 kinases (Swe1 in *S. cerevisiae*) and the opposite effect of the Cdc25 phosphatases (Mih1 in *S. cerevisiae*) [7]. Budding yeast is an exception because this biochemical switch does not play a role in replication stress or DNA damage-induced cell cycle arrest. Instead, this control is the basis of the morphogenesis checkpoint, a mechanism that delays the mitotic activation of Cdc28 in response to many environmental stresses that provoke a transient depolarization of the actin cytoskeleton, which affects bud construction [8,9]. However, more recent observations have also connected Swe1 regulation to some aspects of the response to interrupted DNA synthesis. Swe1 accumulates in hydroxyurea-treated cells in a DNA-damage checkpoint independent manner preventing Cdc28-associated mitotic activities. Later on Swe1 degradation is required for proper recovery from hydroxyurea-induced arrest [10]. Swe1 degradation is triggered by the Mec1-Rad53 DNA-damage checkpoint cascade and plays also a crucial role in the control of morphogenetic events during DNA replication stress [11]. In particular, the DNA-damage checkpoint triggers the switch from apical to isotropic bud growth to maintain proper bud morphogenesis. Actin cytoskeleton dynamics along the cell cycle is controlled by different cyclin-Cdc28 kinases [12,13]. The switch from apical to isotropic bud growth requires activation of the mitotic Clb1,2-Cdc28 kinases in the G2 phase. During the response to DNA damage, Mec1-Rad53 activation causes Swe1 degradation, which allows to build up Clb1,2-Cdc28 activity to switch off apical bud growth.

Mitogen-activated protein kinases (MAPK) are at the core of many signal transduction pathways, orchestrating specific cellular responses to a wide range of stimuli [14]. *S.cerevisiae *cells contain five MAPK that regulate mating (Fus3), pseudohyphal/invasive growth (Kss1), sporulation (Smk1), response to high osmolarity (Hog1) and response to cell wall stress (Slt2) [15]. Slt2 is the MAPK of the cell wall integrity pathway [16,17]. Slt2 is a functional homolog of human ERK5 [18], a MAPK that is activated in response to not only growth factors, but also physical and chemical stresses [19,20]. In yeast, Slt2 is activated under conditions that stress the cell surface, such as growth at high temperatures, hypo-osmotic shock, polarized growth, actin perturbation, or the presence of compounds or mutations that interfere with cell wall biosynthesis [21]. Once activated, Slt2 controls the expression of the genes involved in cell wall biosynthesis through the regulation of transcription factors Rlm1 and SBF (Swi4-Swi6) to maintain cell integrity [22-24]. In addition to gene expression control, Slt2 is also related to the regulation of actin cytoskeleton polarization [25] and contributes, although partially, to the mitotic delay induced by Ca^2+ ^or actin cytoskeleton perturbation as part of the morphogenesis checkpoint mechanism [26,27]. Slt2 is also involved in the cellular response to oxidative stress through the control of cyclin C degradation [28]. Cross-talks between MAPK pathways are common. Thus, the Hog1 kinase (the functional homolog of mammalian p38 MAPK) has been recently described to operate with Slt2 in the adaptation to zymolyase-mediated cell wall stress [29].

MAPKs have been related to the response to DNA damage. Mammalian MAPKs are grouped into the ERK, JNK/SAPK and p38 families [30,31]. Different genotoxic treatments activate p38, which contributes to the establishment of cell cycle checkpoints [32]. Activation of p38 involves the ATM/ATR checkpoint pathway, along with other mechanisms which are yet to be established. Activation of ERK and JNK kinases is also induced by multiple DNA damage stimuli [33-38]. Moreover, both ERK1 and ERK2 kinases are required for the proper checkpoint activation by facilitating activation of ATM and ATR [39-42]. Regarding *S. cerevisiae*, we previously showed that Slt2 is activated by hydroxyurea and that the *slt2 *mutant is sensitive to this drug [43]. Furthermore, genetic interactions have connected Slt2 to DNA-damage checkpoint proteins and the response to MMS [43,44]. Here, we extend our work by carrying out a comprehensive analysis of the connection of Slt2 MAPK with the cellular response to different types of DNA damage caused by a wide range of genotoxic agents.

## Results

### The *slt2 *mutant strain is hypersensitive to genotoxic agents

**Figure 1:**
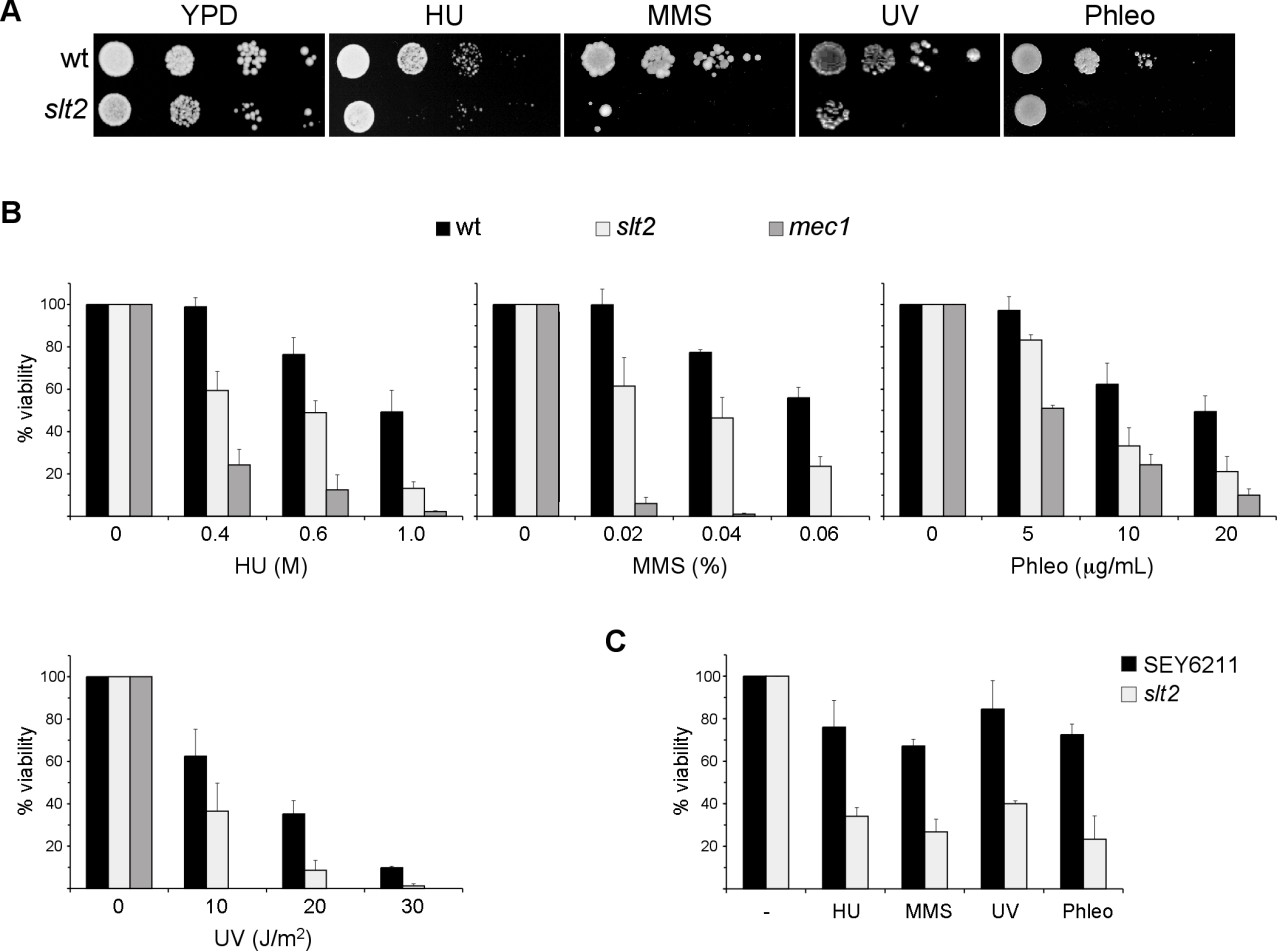
**Hypersensitivity of *slt2 *mutant strain to genotoxic stress**. A) 10-fold serial dilutions from exponentially growing cultures of wild type (W303-1a) and *slt2 *(JCY1062) strains were spotted onto YPD medium containing 100 mM hydroxyurea, 0.025% MMS or 5 μg/mL phleomycin or were exposed to UV radiation (35 J/m^2^). Plates were incubated at 25°C for 3 days. B) Aliquots from exponentially growing cultures of wild type (W303-1a), *slt2 *(JCY1062) and *mec1 sml1 *(JCY1039) strains were incubated for 90 min. at the indicated doses of HU, MMS and phleomycin or were exposed to different doses of UV radiation. Cells were plated on YPD and the percentage of surviving cells relative to untreated controls was determined. C) Aliquots from exponentially growing cultures of wild type (SEY6211) and *slt2 *(JCY193) strains were incubated in the presence of 0.4 M HU, 0.02% MMS, 5 μg/mL phleomycin for 1 hour or were exposed to UV radiation (10 J/m^2^). Cell survival relative to untreated controls was determined.

Cells must cope with different genotoxic stresses to guarantee genomic integrity. The nature and form of action of these genotoxic stresses notably differ. Treatment with hydroxyurea (HU) inhibits ribonucleotide reductase, causing a depletion of dNTP pools, which interferes with DNA replication fork progression and originates subsequent chromosome breakages. Previous work from our group demonstrated that *slt2 *mutant strain growth in the presence of HU is severely affected [43]. We wondered whether Slt2 could also be related to other types of DNA damage apart from replication blockage. To investigate this possibility, *slt2 *mutant strain growth was assayed under conditions that induce the methylation of bases (incubation with alkylating agent methylmetanosulfonate -MMS-), the covalent cross-linking of adjacent pyrimidine bases (irradiation with UV light) or double-strand breaks (incubation with phleomycin). As Figure 1A illustrates, the *slt2 *mutant strain was unable to properly grow when compared to the wild-type strain, not only in the presence of HU, but also in the presence of MMS, phleomycin, or even after UV irradiation. Quantitative survival assays with various doses of genotoxic treatments confirmed an increased loss of cell viability in the absence of Slt2 (Figure 1B). The sensitivity of *slt2 *cells to genotoxic stress was less severe than the one observed in the DNA-damage checkpoint mutant *mec1 *(Figure 1B). The original W303-1a strain contains the *rad5-535 *mutation, which could contribute to the observed growth defects. Therefore, growth analysis was also carried out in a *RAD5 *independent genetic background. As it is shown in Figure 1C, Slt2 inactivation in the SEY6211 strain also originated a reduced cell viability. All these results indicate that yeast cells need a functional Slt2 MAP kinase to optimally survive DNA damage, whatever the nature of the damage, suggesting that Slt2 plays a central role in the cellular response to genotoxic stress.

### Slt2 is activated by genotoxic stresses

**Figure 2:**
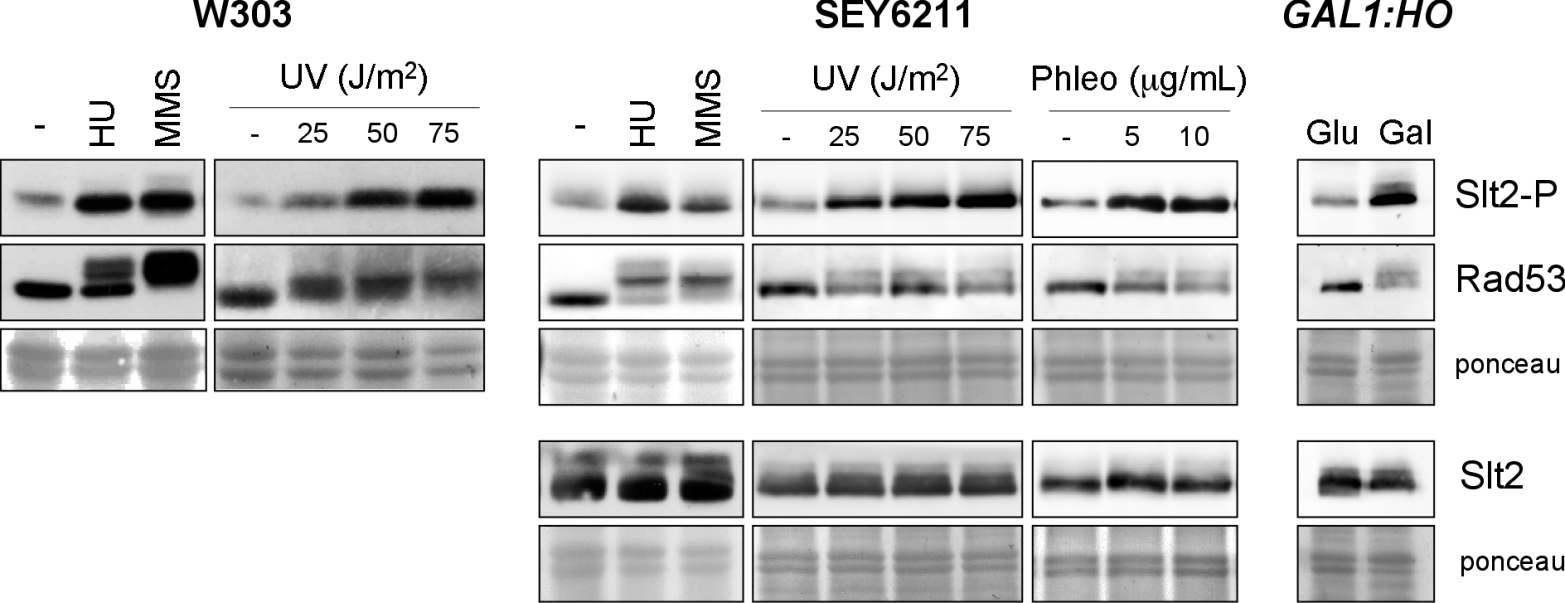
**Activation of Slt2 MAP kinase by genotoxic stress**. Exponentially growing cultures of the wild type W303-1a and SEY6211 strains were split and incubated for 60 min in the absence or presence of 200 mM hydroxyurea, 0.04% MMS, 5 and 10 μg/mL phleomycin, or were irradiated with different doses of UV radiation as indicated. Cultures of the *GAL1:HO *(JKM139) mutant strain grown on rafinose were split and incubated for 4 hours after the addition of glucose or galactose up to 2%. The level of Slt2 phosphorylated in the activation loop and total Slt2 protein in cell extracts was determined by western analysis. Analysis of chekpoint kinase Rad53 activation is shown as a control of the presence of genotoxic stress. Ponceau staining of the membranes are shown as loading control.

**Figure 4:**
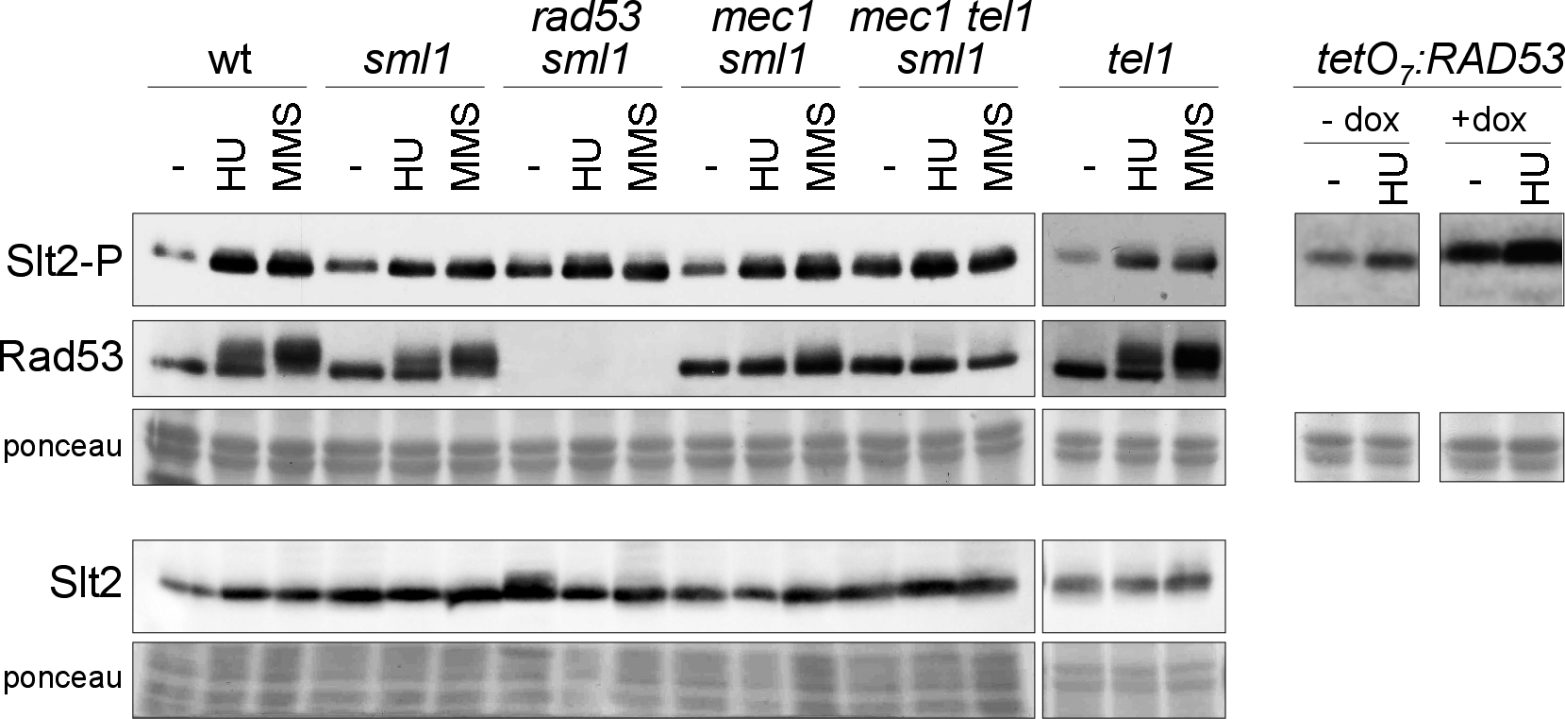
**Activation of Slt2 by genotoxic stress in DNA integrity checkpoint mutants**. Exponentially growing cultures of the wild type (W303-1a), *sml1 *(JCY1144), *rad53 sml1 *(JCY1038), *mec1 sml1 *(JCY1039), *mec1 tel1 sml1 *(JCY1275) and *tel1 *(JCY1258) strains were split and incubated for 60 min in the absence or presence of 200 mM hydroxyurea or 0.04% MMS. Exponentially growing cultures of the *tetO_7_:RAD53 *(JCY1149) strain were incubated for 6 hours in the absence or presence of 5 μg/mL doxicycline in order to repress the *tetO_7 _*promoter followed by 60 min incubation in the absence or presence of 200 mM hydroxyurea. The level of phosphorylated Slt2, total Slt2 protein and the chekpoint kinase Rad53 was determined by western analysis. The ponceau staining of the membranes are shown as loading control.

**Figure 5:**
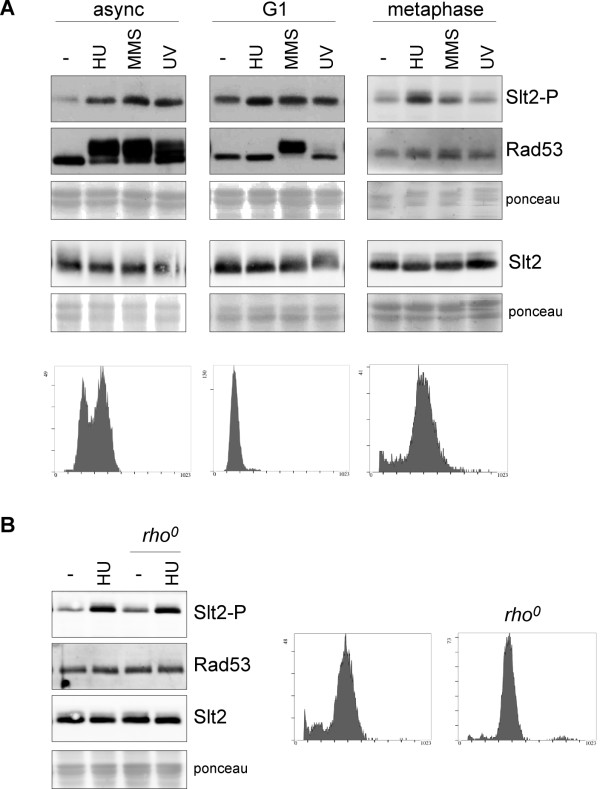
**Analysis of the cell cycle dependent activation of Slt2 by genotoxic stress**. A) Exponentially growing cells of the wild type strain (W303-1a) were arrested in G1 by incubation in the presence of 5 μg/mL α-factor for 2 hours. Exponentially growing cells of the *GAL1:CDC20 *strain (JCY1645) were arrested in metaphase by incubation in YPD medium for 3 hours. Cell cycle arrest was confirmed by cell morphology (more than 95% of unbudded cells or more than 90% of large budded cells respectively) and analysis of DNA content by flow cytometry (lower panels). Once arrested, cells were incubated for 60 min in the absence or presence of 200 mM hydroxyurea or 0,04% MMS, or were exposed to UV radiation (50 J/m^2^), while maintaing cell cycle arrest with α-factor or glucose. In the case of α-factor arrested cells, 600 mM HU, 0.12% MMS and 150 J/m^2 ^UV radiation was used. The level of phosphorylated Slt2, total Slt2 protein and the chekpoint kinase Rad53 was determined by western analysis. The ponceau staining of the membranes are shown as loading control. B) Slt2 activation by HU in metaphase arrested cells of the *GAL1:CDC20 *strain (JCY1645) and a *rho^0 ^*strain derived from it were analysed as described in A.

Slt2 is activated by phosphorylation in the activation loop. In previous works, we observed a dramatic increase in the phosphorylation state of the Slt2 MAP kinase after addition of hydroxyurea [43]. Here, we extended the analysis by testing whether other types of DNA damage also cause Slt2 activation. First, W303-1a cells were treated with HU or MMS, or were irradiated with different UV doses to induce DNA damage. Occurrence of damage was monitored by analyzing the phosphorylation state of the checkpoint kinase Rad53. The appearance of lower Rad53 electrophoretic mobility bands corresponding to the phosphorylated protein confirmed that the checkpoint was activated by these treatments. It is interesting to note that higher levels of phosphorylated Slt2 were detected in these cells, indicating that the MAP kinase Slt2 is activated in those cells incubated in the presence of HU or MMS, or those exposed to UV radiation (Figure 2). A similar result was obtained in a W303-derived *RAD5 *strain and in the SEY6211 genetic background (Figure 2). Slt2 activation was also observed after induction of double-strand breaks (DSB) with phleomycin. Next, we analyzed the response to a single DSB induced by the addition of galactose to raffinose-grown cells expressing the HO endonclease under *GAL1 *promoter control. As Figure 2 depicts the levels of phosphorylated Slt2 drastically increased after the induction of a single DSB. This in not due to the change in carbon source since Slt2 activation after addition of galactose is not observed in a wild type control strain. All these observations are consistent with the above-described *slt2 *mutant hypersensitivity to genotoxic stresses and indicate that Slt2 activation is a crucial step in the cellular response to all kinds of DNA damage. Interestingly, Slt2 activation by genetic stresses is mostly, if not totally, mediated by a post-translational mechanism since Slt2 protein level is not significantly affected (Figure 2, 4 and 5).

**Figure 3:**
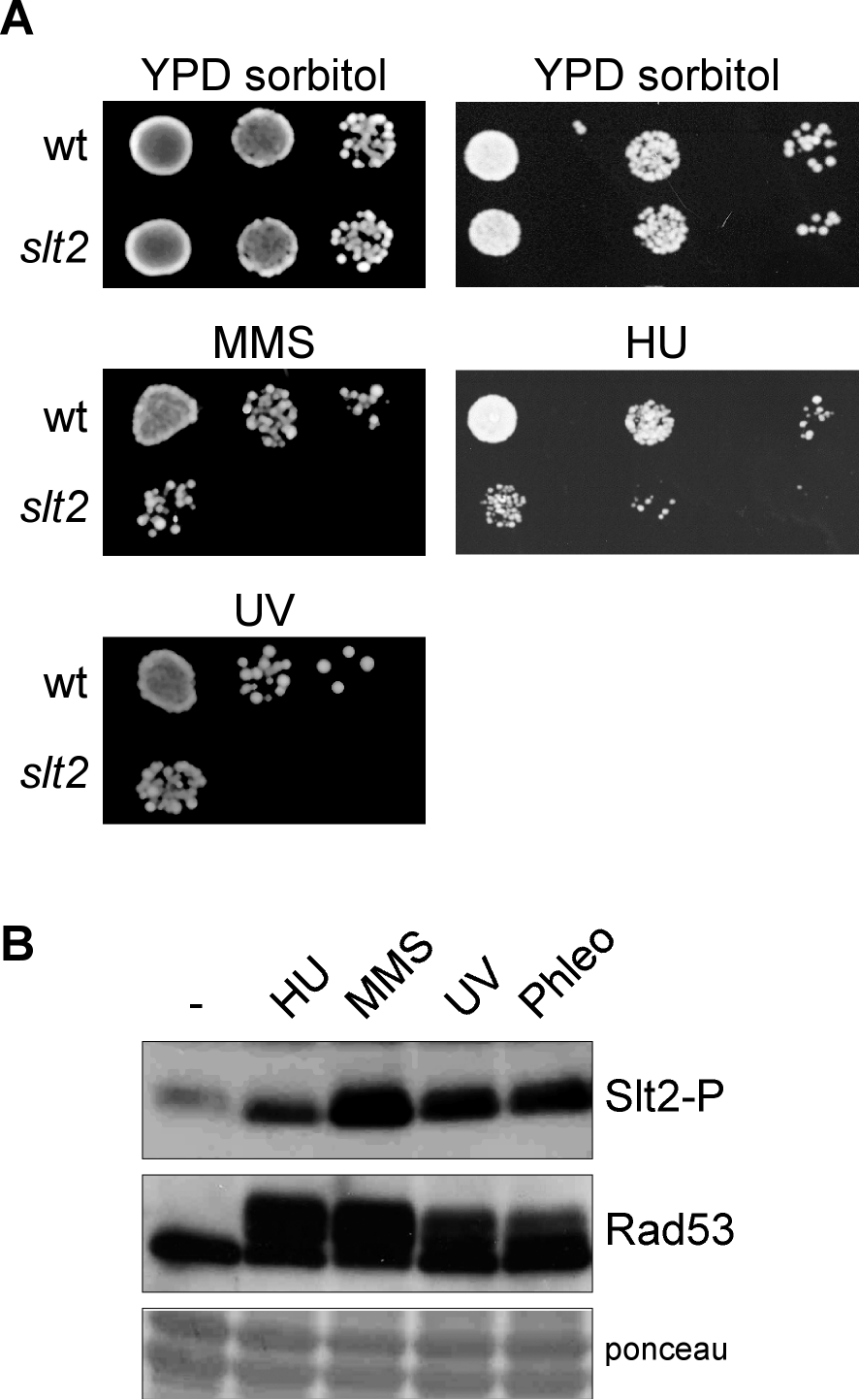
**Analysis of the hypersensitivity of *slt2 *cells to genotoxic stress and the activation of Slt2 by genotoxic stress in osmotically supported medium**. A) 10-fold serial dilutions from exponentially growing cultures of wild type (W303-1a) and *slt2 *(JCY1062) strains were spotted onto YPD medium containing 1 M sorbitol and 200 mM hydroxyurea or 0.03% MMS or were exposed to UV radiation (40 J/m^2^). Plates were incubated at 25°C for 3 days. B) Cultures of the wild type W303-1a strain grown on YPD containing 1 M sorbitol were split and incubated for 60 min in the absence or presence of 200 mM hydroxyurea, 0.04% MMS or 5 μg/mL phleomycin, or were irradiated with UV radiation (50 J/m^2^). The level of phosphorylated Slt2 and the chekpoint kinase Rad53 (as a control of the presence of genotoxic stress) was determined by western analysis. The ponceau staining of the membrane is shown as loading control.

Slt2 is involved in cell wall biosynthesis. It is activated by cell surface stress to maintain cell integrity. To investigate whether the activation of Slt2 by genotoxic stresses is a direct response to damage or an indirect effect caused by the morphogenetic stress deriving from genotoxic treatments, we repeated the experiments in cells grown in the presence of an osmotically stabilized agent (sorbitol). The results showed that both the hypersensitivity of slt2 cells to (Figure 3A) and Slt2 activation by (Figure 3B) HU, MMS, phleomycin and UV radiation also occur in the presence of sorbitol. These results further reinforce a direct connection of Slt2 to the DNA damage response.

### Analysis of Slt2 activation in DNA-damage checkpoint mutants

The cellular response to genotoxic stress is governed by the DNA integrity checkpoint pathway. We wondered whether Slt2 activation by genotoxic stresses was mediated by the DNA damage checkpoint. To investigate this, activation of Slt2 by HU or MMS was analyzed in the mutant strains in checkpoint upstream kinases Mec1 and Tel1 or in the effector kinase Rad53 (Figure 4). Rad53 and Mec1 are essential genes so we used in these cases strains containing the *sml1 *mutation, which is known to suppress *rad53 *and *mec1 *lethality. It is noteworthy that strong Slt2 activation took place in the absence of genotoxic agents in *rad53 *and *mec1 tel1 *mutant strains. This is in agreement with previously reported results and is probably a response to the cell morphology and cell wall defects characteristic of these checkpoint kinase mutants [11]. Another important aspect is that incubation with HU or MMS brought about higher levels of activated Slt2 in the *tel1, mec1, mec1 tel1 *or *rad53 *mutant cells. A similar result was obtained with the *tetO_7_:RAD53 *mutant strain. These results demonstrate that Slt2 activation by genotoxic stress is not mediated by the DNA damage checkpoint.

### Slt2 is activated by HU in post-replicative cells

As the response to genotoxic stress varies depending on the cell cycle stage [45-48], we wondered whether Slt2 activation in response to genotoxic agents depends on the cell cycle stage. Accordingly, Slt2 activation by HU, MMS and UV radiation was analyzed in cells arrested in G1 with α-factor and cells arrested in G2/M by inactivating the *CDC20 *gene (Figure 5A). A mild Slt2 activation was observed in G1 cells treated with HU. By contrast, no significant increase in phosphorylated Slt2 due to incubation of cells with MMS or UV irradiation was detected in G1 cells; however, it has to be noted that α-factor caused Slt2 activation, which could preclude genotoxic-induced Slt2 activation. In G2/M cells, no activation was observed in the presence of MMS or after UV irradiation in contrast to what was detected in cycling cells. These results suggest that Slt2 activation by MMS or UV radiation probably occurs during the S phase. On the contrary, Slt2 was activated by HU in G2/M cells. We wondered whether Slt2 activation by HU could be related to mitochondrial DNA replication; however, the fact that Slt2 activation is also observed in a *rho^0 ^*derived strain ruled out this possibility (Figure 5B). HU is an inhibitor of ribonucleotide reductase, which catalyzes the limiting step in dNTP biosynthesis. Incubation of cells with HU causes a reduction of dNTP pools and a consequent blockage of S phase progression. The fact that HU affects Slt2 activity in post-replicative cells suggests that Slt2 activation could be, at least in part, a direct response to an alteration of the nucleotide pools, which also could indicate that Slt2 might be involved in the control of dNTP pools.

### Analysis of dNTP pools in the absence of Slt2

**Figure 6:**
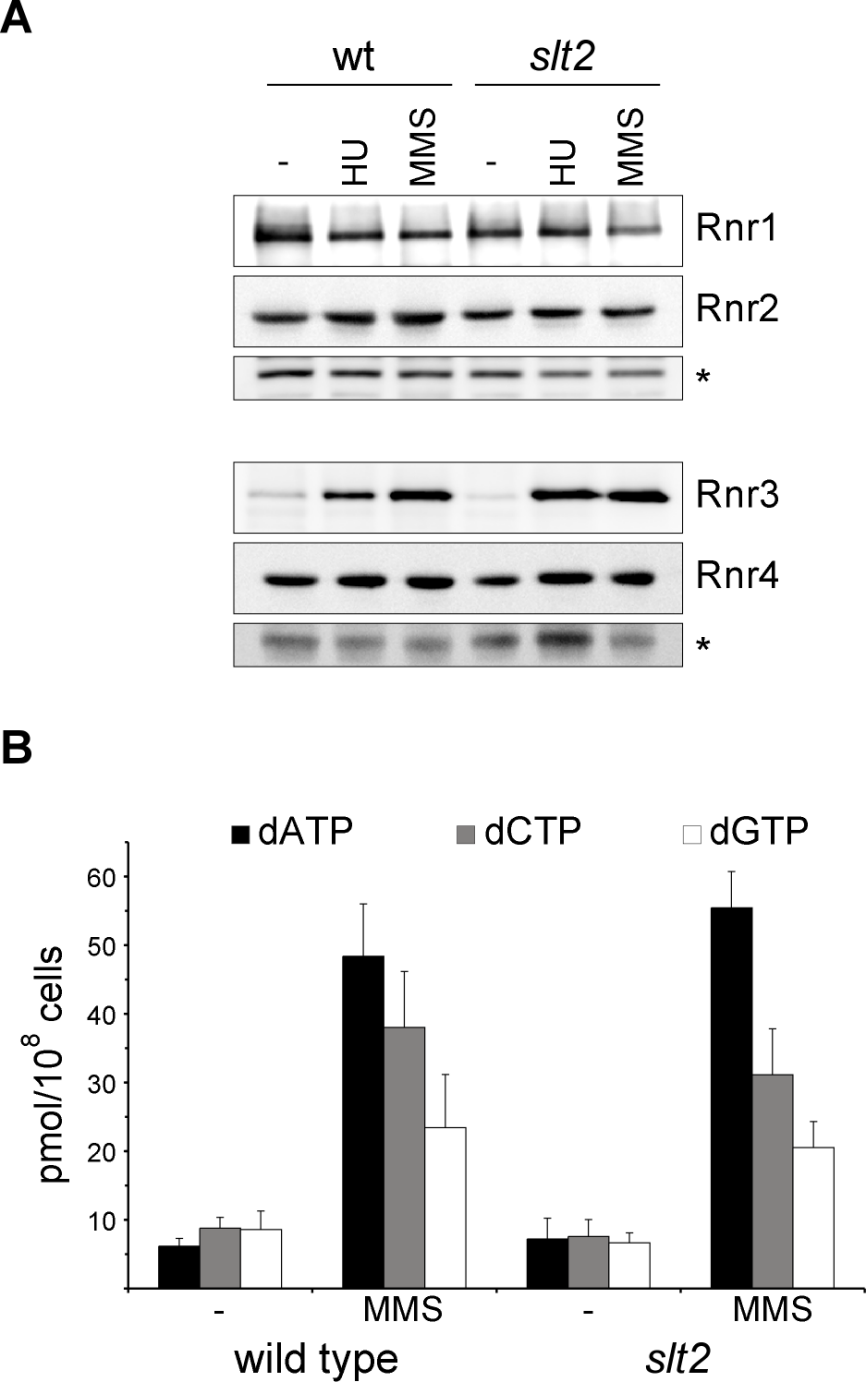
**Analysis of ribonucleotide reductase (RNR) function in the *slt2 *mutant strain**. A) Exponentially growing cultures of the wild type W303-1a strain were split and incubated for 120 min in the absence or presence of 200 mM hydroxyurea or 0.04% MMS. The protein level of the different subunits of the RNR enzyme was analyzed by western blot. A non-specific band labelled with an asterisk that cross-react with the antibody is shown as loading control. B) Exponentially growing cultures of the wild type W303-1a strain were split and incubated for 120 min in the absence or presence of 0,04% MMS. The cellular content of dATP, dCTP and dGTP were determined in crude cell extracts. Similar results were obtained with cells incubated for 60 min in the presence of HU or MMS.

In an initial approach to characterize whether Slt2 could affect dNTP pools, we first analyzed the ribonucleotide reductase protein levels in *slt2 *cells in normal conditions or after induction of DNA damage. A Western blot analysis revealed that all the ribonucleotide reductase subunits were expressed at a similar level in wild-type and *slt2 *cells both before and after HU or MMS treatments (Figure 6A). It is possible that ribonucleotide reductase activity could be defective in *slt2 *mutant cells despite the amount of ribonucleotide reductase enzyme not being altered. To test this, we measured the cellular content of dATP, dCTP and dGTP in the wild-type and *slt2 *mutant strains. As observed in Figure 6B, inactivation of Slt2 caused no significant changes in the concentration of the three dNTPs under both basal conditions and in MMS-treated cells. This result demonstrates that Slt2 is not involved in the control of dNTP pools.

### Analysis of DNA-integrity checkpoint activation in the *slt2 *mutant strain

**Figure 7:**
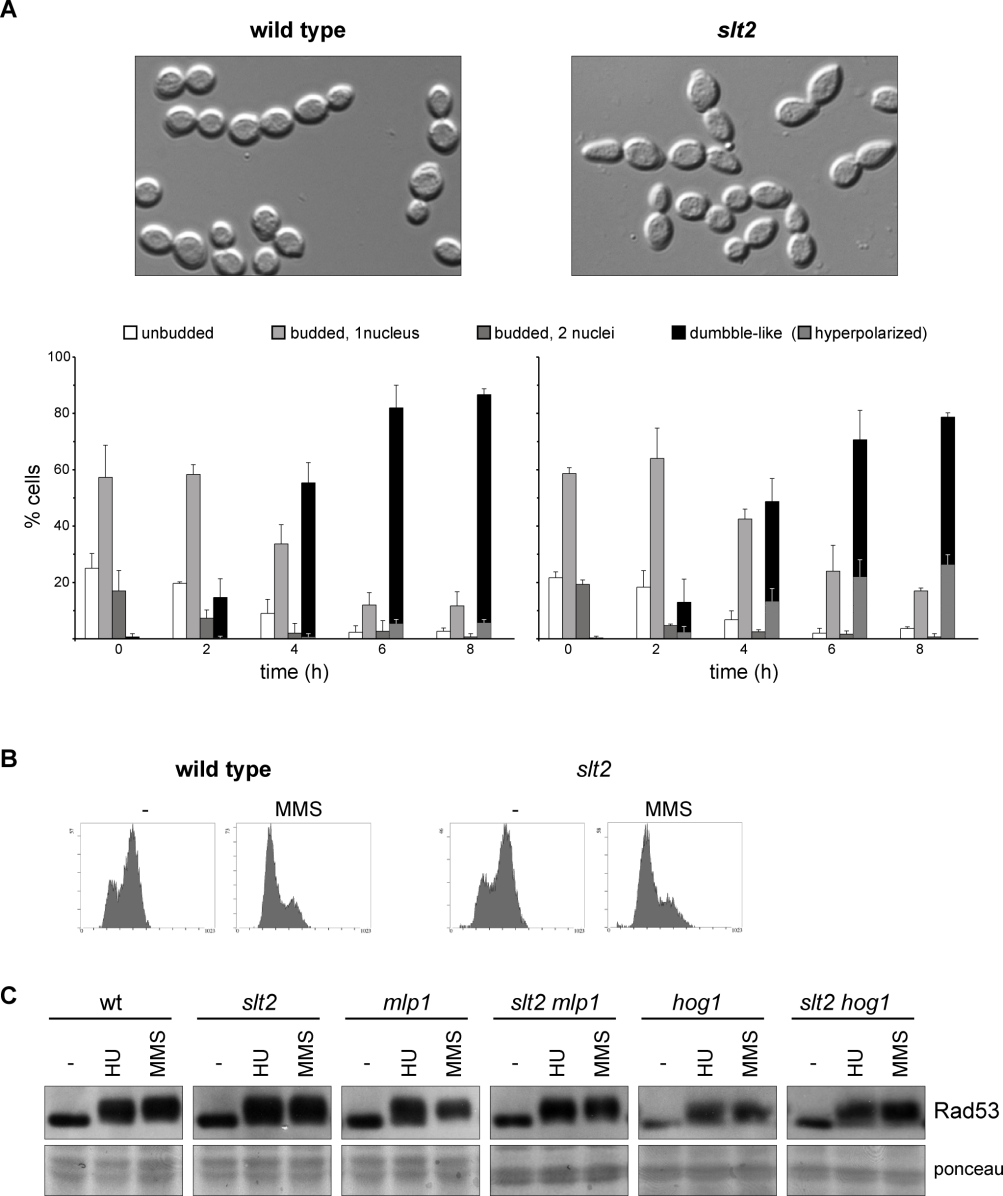
**Functional analysis of the DNA integrity checkpoint in the *slt2 *mutant strain**. A) Exponentially growing cultures of the wild type (W303-1a) and *slt2 *(JCY1062) strains were split and incubated for the indicated time in the absence or presence of 200 mM hydroxyurea. Cell cycle distribution of cells was analyzed determining cellular morphology and number of nuclei by microscopy. Cells were classified as unbudded, budded with a single nucleus, budded with two nucleus and dumbble-like cells (cells with a large bud similar in size to mother cells but with a single nucleus), which are indicative of G2/M cell cycle arrest. The presence of dumbble cells with an abnormal elongated bud is also indicated. Graphs represent cell distribution derived from at least three independent cultures. No change in cell distribution was observed in untreated controls at 6 hours incubation time. Pictures correspond to the HU-treated sample at 8 hours incubation time. B) Analysis of the DNA content by flow cytometry of samples from exponentially growing cultures of the wild type (W303-1a) and *slt2 *(JCY1062) strains incubated for 60 min in the absence or presence of 0.04% MMS. C) Exponentially growing cultures of the wild type (W303-1a), *slt2 *(JCY1062), *mlp1 *(JCY1334), *slt2 mlp1 *(JCY1336), *hog1 *(JCY1489) and *slt2 hog1 *(JCY1606) strains were split and incubated for 60 min in the absence or presence of 200 mM hydroxyurea or 0,04% MMS. Activation of the chekpoint kinase Rad53 was determined by western analysis. The ponceau staining of the membrane is shown as loading control.

In mammalian cells, p38 and ERK1,2 MAPKs are involved in establishing the cell cycle checkpoint after DNA damage [32,39-42]. Accordingly, we investigated whether MAPK Slt2 was required to arrest cell cycle progression after the induction of a replicative stress with HU. After 6 hours, wild-type cells were blocked in the G2/M phase, as deduced from the accumulation of dumbbell cells characterized by a large bud similar in size to the mother cell and a single nucleus close to the bud neck. As seen in Figure 7A, the *slt2 *mutant strain also accumulated nearly 80% of the large-budded cells, similarly to what observed in the wild-type strain. Nevertheless, it is noteworthy that a significant amount of the arrested cells have somewhat elongated buds (26.3 ± 3.5% in *slt2 *compared to 5.6 ± 1.2% in the wild-type). These observations indicate that Slt2 is not required for HU-induced cell cycle arrest, but is involved in maintaining proper bud morphogenesis after DNA damage.

We also analyzed cell cycle arrest after inducing DNA damage with MMS. In this case, there was no homogenous terminal morphology, but cell cycle arrest was revealed by the DNA content analysis, showing a clear accumulation of cells with non-replicated DNA after a 1-hour incubation in the presence of MMS. Importantly, this accumulation was observed in both the wild-type and the *slt2 *mutant strains (Figure 7B). Thus, MMS-induced cell cycle arrest occurs in the absence of Slt2.

Finally, we also investigated whether checkpoint activation normally occurs in the absence of Slt2. To test this, *slt2 *mutant cells were subjected to replicative stress or DNA damage by incubation with HU or MMS, respectively, and the presence of phosphorylated Rad53, as indicative of checkpoint activation, was analysed by Western blot. The results showed that phosphorylated Rad53 accumulated at similar levels in the wild-type and *slt2 *mutant strains after genotoxic treatments (Figure 7C). Thus, the DNA damage checkpoint is functional in the absence of Slt2, at least until the Rad53 activation step.

Slt2 has a pseudo-kinase paralog in yeast, protein Mlp1. Mlp1 shares a function with Slt2 in transcriptional activation [23]. Therefore, it is possible that Mlp1 could be functionally redundant with Slt2, and that it could activate the DNA integrity checkpoint in the absence of Slt2. However, we detected a proper activation of Rad53 by HU and MMS in the *mlp1 *and *slt2 mlp1 *mutant strains (Figure 7C). This observation confirms that Slt2 kinase and its relative Mlp1 protein are not required for proper Rad53 activation.

Increasing evidence indicates there are cross-talks between the MAPK cascades in yeast. Hog1, the MAPK involved in the response to osmotic stress, is especially interesting because recent works have related the Slt2 and Hog1 functions in the activation of the cell wall gene expression [29]. Furthermore, Hog1 is the yeast homolog to mammalian p38 MAPK. As mentioned above, p38 plays an important role in cell cycle checkpoints in response to DNA damage. Therefore, we investigated whether Hog1 was involved in Rad53 activation. However, this was not the case because phosphorylated Rad53 normally accumulated after HU and MMS treatments in the absence of Hog1 (Figure 7C). Moreover, no defect in Rad53 activation was detected in a *slt2 hog1 *double mutant, which ruled out any functional redundancy between Slt2 and Hog1 in checkpoint activation.

### Slt2 is required for the proper degradation of Swe1 after DNA damage

**Figure 8:**
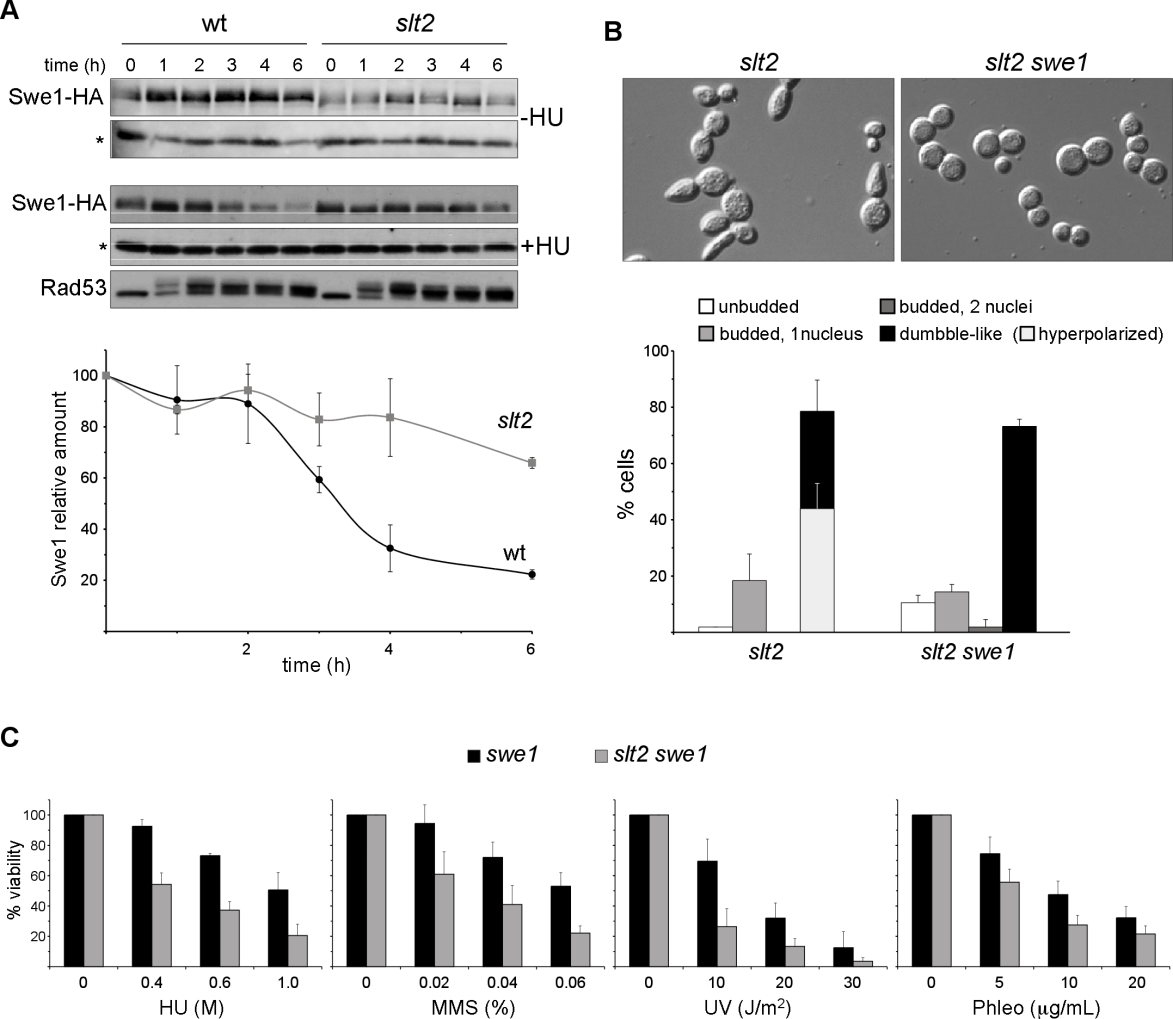
**Analysis of Swe1 protein level in *slt2 *mutant cells after a replicative stress**. A) Exponentially growing cells of the wild type (JCY1316) and *slt2 *(JCY1318) strains expressing a HA-epitope tagged Swe1 protein were incubated in the absence or presence of 200 mM hydroxyurea. Swe1 protein level and activation of the chekpoint kinase Rad53 was analyzed at the indicated time after the addition of HU by western blot. A non-specific band labelled with an asterisk that cross-react with the antibody is shown as loading control. Graph represents the relative amount of Swe1 protein related to the non-specific band in the HU-treated cells derived from three independent assays. B) Exponentially growing cultures of the *slt2 *(JCY1062) and the *slt2 swe1 *(JCY1633) strains were incubated in the presence of 200 mM hydroxyurea for 6 hours. Cell cycle distribution of cells and the presence of abnormal elongated bud were analyzed as described in Figure 7. Graphs represent cell distribution derived from at least three independent cultures. C) Aliquots from exponentially growing cultures of the *swe1 *(JCY1632) and the *slt2 swe1 *(JCY1633) strains were incubated for 90 min. at the indicated doses of HU, MMS and phleomycin or were exposed to different doses of UV radiation. Cells were plated on YPD and the percentage of surviving cells relative to untreated controls was determined.

Recently, a morphogenetic function for the DNA integrity checkpoint has been described, which consists in switching off bud apical growth after damage [11]. This is achieved by the degradation of CDK inhibitor kinase Swe1. Cells with a defective checkpoint are unable to degrade Swe1 and as a consequence, they cannot induce the switch from polar to isotropic bud growth, resulting in the formation of elongated buds. By considering that Slt2 has been related to Swe1 regulation [26,27] and that *slt2 *mutant cells manifested a hyperpolarization defect in response to DNA damage, we wondered whether Slt2 is required for the morphogenetic response controlled by the Rad53 checkpoint kinase. To investigate this, the Swe1 protein level was analyzed after incubating cells with HU (Figure 8). As previously described, Swe1 is eliminated from wild-type cells after genotoxic stress to reduce to less than 20% of the initial protein level after 6 hours. It is remarkable to note that Swe1 protein decay was minimized in the absence of Slt2, and that more than 50% of the initial protein remained after 6 hours. This was not caused by cell cycle effects, as cell cycle distribution of *slt2 *mutant was roughly similar to that of wild type strain (see Figure 7A) neither to differences in checkpoint activation as Rad53 phosphorylation occurred with similar kinetics. This result indicates that Slt2 is involved in the morphogenetic response after DNA damage and is required for optimal Swe1 degradation in response to DNA damage. Interestingly, hyperpolarization of *slt2 *mutant cells is not observed when Swe1 kinase is inactivated (Figure 8B). This demonstrates that Slt2 control of bud morphogenesis in response to DNA damage is mediated by the Swe1 kinase. However, Slt2 inactivation caused loss of cell viability even in the absence of Swe1 (Figure 8C), indicating that the hipersensitivity to genotoxic stresses involves additional Swe1-independent mechanisms.

## Discussion

Activation of cell-cycle checkpoints in response to various types of DNA damage is essential for the maintenance of genomic stability in eukaryotic cells. This work describes how the Slt2 MAP kinase is activated in response to DNA damage and that Slt2 is essential to properly cope with genotoxic stresses. Slt2 is involved in cell wall assembly and is activated by cell wall damage, so it could be possible that *slt2 *mutant hypersensitivity to genotoxic treatments or Slt2 activation simply result from increased cell wall permeability or unknown cell wall damage caused by the treatments. This possibility is unlikely, however, because hypersensitivity to genotoxic treatments is also observed in the presence of sorbitol, which indicates that cell death is not related to cell wall defects. Most remarkably, Slt2 activation after induction of a single DSB in the *GAL1:HO *strain, a process that has a specific effect on DNA integrity and is not related to cell wall, strongly supports a genuine role for Slt2 in the response to genotoxic stress. This conclusion is also reinforced by a recent genetic interaction network analysis that connected Slt2 to the cellular response to MMS [44]. It is important to note that both Slt2 activation and hypersensitivity of the *slt2 *mutant are observed in response to a wide range of genotoxic stresses, which result in different types of DNA damage: e.g., DSB, thymidine dimers, nucleotide alkylation or replicative forks stalling. This reinforces the importance of Slt2 in the response to genotoxic stresses and suggests that Slt2 could play a common role in the response to different types of damage.

Response to DNA damage is primarily governed by the activation of the DNA-integrity checkpoints. However, Slt2 is activated even in the absence of a functional checkpoint, indicating that genotoxic stress is transduced to the Slt2 kinase by a checkpoint-independent mechanism. In fact, Slt2 activation and Rad53 activation after inducing DNA damage occur independently of each other. Thus, both kinases appear to fulfill complementary functions necessary for cell survival in parallel independent DNA-damage signaling pathways. This indicates that the response of yeast cells to genotoxic stresses could be more complex than previously suspected, and may involve new crucial players like Slt2 MAPK. In mammalian cells, p38 MAPK activation in response to DNA damage has been reported to be dependent on ATM/ATR checkpoint kinases in some cases; however, in addition to the checkpoint pathway, other mechanisms still to be established activate p38 MAPK in response to DNA damage [32]. Thus, a mechanism directly linking MAPK activation to DNA damage could be conserved in eukaryotic cells.

It is not clear how genotoxic stresses could activate Slt2. We have observed that upregulation of Slt2 is mostly, if not totally, mediated by a post-translational mechanism. As commented above, Slt2 activation occurs in osmotically supported cells or in *cdc42 *mutant cells that cannot bud [43] and it was observed after specific induction of a single DSB, which strongly suggests that Slt2 activation is not an indirect effect. The observed Slt2 activation could be a direct result of DNA lesions. Remarkably, however, Slt2 activation by MMS and UV is cell cycle-regulated since it does not occur in the cells arrested in G2/M. This suggests that Slt2 does not respond to primary damage and should be related to alterations that appear as damaged cells progress through the cell cycle.

Activation by HU seems to be a different case since HU induced Slt2 activation, even in G2/M-arrested cells. It could be expected that HU stress would only affect cells that were actively replicating DNA. The fact that HU induced activation is also observed in post-replicative cells, i.e., cells that do not consume dNTP pools, suggests that Slt2 could respond directly to the inhibition of ribonucleotide reductase activity and the reduction in dNTP pool levels. Very recently, Slt2 has been suggested to repress the expression of the *RNR1*,2,3 genes, as deduced from the increase in mRNAs observed in the *slt2 *mutant strain [44]. However, we detected no change in the protein level in any ribonucleotide reductase subunit after Slt2 inactivation in both untreated and MMS-treated cells. More importantly, dNTP pools, which reflect the physiological function of ribonucleotide reductase activity, were not altered in the *slt2 *mutant under any analyzed condition. Thus, our results argue against a role of Slt2 in controlling ribonucleotide reductase and dNTP pools.

Our findings show that Slt2 plays an important part in the general cellular response to genotoxic stress. Slt2 could mediate a defensive mechanism developed by yeast cells to overcome treatments affecting DNA integrity. Primary cellular defense to DNA damage involves cell cycle arrest to facilitate DNA repair prior to progression through the S phase or mitosis. It is known that Slt2 contributes to the delay of the G2/M transition in response to actin stress [26,27]. However, this is not the case in the response to genotoxic stress since cell cycle arrest occurs in the absence of Slt2. Alternatively, it is tempting to speculate that Slt2 could directly favour DNA damage repair. On the other hand, involvement of Slt2 in the response to genotoxic stress could be related with its known function in cell morphogenesis, the actin cytoskeleton and cell integrity. Although this idea may seem surprising, there is increasing evidence for a direct cross-talk between the DNA checkpoint pathway and cellular morphogenesis. Thus, various checkpoint proteins contribute to cell wall architecture and maintenance of cell polarity [11], Cdk1 regulates Rad53 to orchestrate cellular morphogenesis during cell cycle [49], Rad53 interacts with septins in the bud neck and directs filamentous differentiation in response to genotoxic stress [50], Rad53 phosphorylates Slt2 to control the Slt2-dependent expression of the cell wall *FKS2 *gene in response to caffeine [51], the Mdt1 protein has partially separable functions in both the cell wall and genome integrity pathways [52], and we have observed a synthetic lethality between *slt2 *and *rad53 *mutations that is suppressed by sorbitol, which suggests that lethality is caused by a morphogenetic defect. In fact, bud morphogenesis control has been described as an output of DNA replication checkpoint activation [11], which clearly demonstrates the link between genome maintenance and cell morphogenesis. We have observed that Slt2 is related to the morphogenetic aspects of the replicative stress response. In budding yeast cells, actin cytoskeleton polarization has to be regulated during DNA damage-induced cell cycle arrest to maintain bud morphogenesis. Thus during the response to DNA damage, checkpoint activation has been described to cause degradation of the Swe1 kinase [11], which is the main protein involved in the morphogenesis checkpoint controlling bud growth [8,9]. This leads to the activation of the Clb1,2-Cdc28 kinases, which are responsible for the switch from apical to isotropic bud growth, an essential switch for proper bud morphogenesis [12,13]. We have observed that Swe1 degradation after replicative stress is partially impaired in the absence of Slt2, causing altered bud morphogenesis in *slt2 *cells. Defects in Swe1 degradation has been related to HU sensitivity [10], which could explain *slt2 *phenotype. However, HU and other genotoxic treatments caused a reduced viability of *slt2 *mutant cells even in the absence of Swe1, which indicates that Slt2 must affect cell viability by a Swe1-independent mechanism. How Slt2 influences Swe1 stability is intriguing, and even more so if we consider that this effect is apparently contradictory to the results previously described in the morphogenesis checkpoint context [26] or in response to Ca^2+ ^[53]. In these cases, Slt2 acts by activating Swe1 or by repressing Mih1 to inhibit Cdc28 kinase activity, whereas in the response to replicative stress, Slt2 seems to act by inactivating Swe1 to induce Cdc28 kinase activity. Elucidating the molecular basis of the Slt2 function in the response to genotoxic stresses will help explain this apparent contradiction and to gain insight into the molecular link between cellular morphogenesis and integrity with genome integrity maintenance.

## Conclusions

Our results support a function of MAPK Slt2 in the maintenance of DNA integrity. Inactivation of Slt2 results in hypersensitivity to many types of genotoxic treatments. Moreover, Slt2 is activated by several genotoxic stresses. These results suggest that Slt2 play an important role in the cellular response to DNA damage. Slt2 activation by MMS and UV, but not HU, requires cell cycle progression. Slt2 is not involved in dNTP pools regulation and is not required for DNA-damage induced cell cycle arrest or checkpoint activation. Nevertheless, Slt2 function is important for bud morphogenesis control and optimal Swe1 degradation under replicative stress. The results described here point to the MAPK Slt2 as a new player in the cellular response to genotoxic stresses.

## Methods

### Strains and growth conditions

The yeast strains used in this study are shown in Table 1. The *slt2::TRP1, sml1::kanMX6, tel1::kanMX6, mlp1::kanMX6, hog1::kanMX4, swe1::kanMX6 *and *SWE1-HA-kanMX6 *cassettes were amplified from pFA6a series plasmids (a gift from Dr. J.R. Pringle) or Euroscarf yeast strains and integrated in the indicated parental strain. The substitution of the *RAD53 *promoter by the *tetO_7 _*promoter was obtained by integrating a DNA fragment amplified from plasmid pCM225 (a gift from E. Herrero). To obtain the JCY1645 strain, a plasmid containing the *GAL1:CDC20 *gene (a gift from E. Queralt) was digested with McsI and integrated in W303-1a. To obtain a *rho^0 ^*strain derived from JCY1645, cells were grown to saturation in the presence of 25 g/mL ethidium bromide and streaked for single colonies. The loss of mitochondrial DNA was checked by the failure to grow on medium containing glycerol as sole carbon source and by fluorescence microscopy analysis of DAPI-stained cells. Cells were grown on standard yeast extract-peptone-dextrose (YPD). For growth assays, 10-fold serial dilutions in growth medium were prepared from exponentially growing culture (usually 2-8 × 10^6 ^cells/mL) of the different strains. 5 μL of each dilution was then spotted onto YPD, YPD supplemented with 100 mM hydroxyurea (HU), 0.025% methyl metanosulfonate (MMS) or 5 μg/mL phleomycin and YPD followed by UV irradiation (35 J/m^2^) using the GS Gene Linker™ UV chamber (Bio-Rad). 1 M sorbitol was added to the growth media when required. For induction of genotoxic stress in liquid cultures, 0.2-0.4 M HU, 0.04% MMS or 5 μg/mL phleomycin was added to exponentially growing cultures or cells were exposed to UV irradiation (50 J/m^2^). For induction of a single DSB the *GAL1:HO *strain was grown overnight on yeast extract-peptone-2% raffinose medium, then 2% galactose (or 2% glucose as a negative control) was added to the medium and cells were incubated for 4 hours. Cell cycle arrest at G1 phase was accomplished by the addition of 5 μg/ml α-factor and incubation for 3 hours,. Cell cycle arrest at G2/M phase was accomplished by growing *GAL1:CDC20 *cells in yeast extract-peptone-2% galactose medium (plus 0.1% glucose in the case of the *rho^0 ^*strain) and transferring them to YPD for 3 hours, previously to the genotoxic treatments. To repress the *tetO_7 _*promoter, doxycicline was added to a final concentration of 5 μg/mL.

**Table 1:** Yeast strains

W303-1a	*MATa ade2-1 trp1-1 leu2-3,112 his3-11,15 ura3-52 can1 rad5-535*
SEY6211	*MATa ade2-101 trp1-Δ902 leu2-3,112 his3-Δ200 ura3-52 RAD5*
JKM139^a^	*MATa ade1-100 trp1-1 leu2-3,112 lys5 ura3-52 trp1::hisG hoΔ hml::ADE1 hmr::ADE1 ade3::GAL-HO RAD5*
JCY193	*slt2::LEU2 *in SEY6211
JCY1038^b^	*rad53::HIS3 sml1-1 *in W303-1a
JCY1039^b^	*mec1::TRP1 sml1::HIS3 *in W303-1a
JCY1062	*slt2::TRP1 *in W303-1a
JCY1144	*sml1::kanMX6 *in W303-1a
JCY1149	*tTR'::LEU2 tetO_7_::RAD53-kanMX4 *in W303-1a
JCY1258	*tel1:: kanMX6 *in W303-1a
JCY1275	*tel1:: kanMX6 *in JCY1039
JCY1316	*SWE1-HA-kanMX6 *in W303-1a
JCY1318	*SWE1-HA-kanMX6 *in JCY1062
JCY1334	*mlp1::kanMX6 *in W303-1a
JCY1336	*mlp1::kanMX6 *in JCY1062
JCY1489	*hog1::kanMX4 *in W303-1a
JCY1616	*hog1::kanMX4 *in JCY1062
JCY1632	*swe1::kanMX6 *in W303-1a
JCY1633	*swe1::kanMX6 *in JCY1062
JCY1645	*GAL1:CDC20-TRP1 *in W303-1a

^a ^from Dr. J.E. Haber; from ^b ^Dr. J. Torres

### Western blot analysis

Approximately 10^8 ^cells were collected, resuspended in 100 μl water and, after adding 100 μl 0.2 M NaOH, they were incubated for 5 min at room temperature. Cells were collected by centrifugation, resuspended in 50 μl sample buffer and incubated for 5 min at 95°C. Extracts were clarified by centrifugation, and equivalent amounts of protein were resolved in an SDS-PAGE gel and transferred onto a nitrocellulose membrane. The primary antibodies used in this study include anti-fosfo-44/42 Map kinasa Thr200/Tyr204 (Cell Signalling) to detect activated Slt2, anti Mpk1-yC20 (Santa Cruz Biotechnology) to detect total Slt2 protein, anti Rad53-YC19 (Santa Cruz Biotechnology), anti-HA 3F10 monoclonal antibody (Roche), and anti Rnr1, Rnr2, Rnr3 and Rnr4 (kindly provided by M. Huang, University of Colorado). Blots were developed with HRP-labeled secondary antibodies using the ECL Advance Western Blotting Detection Kit (GE Healthcare Life Sciences). Bands were quantified with a ImageQuant™ LAS 4000mini biomolecular imager (GE Healthcare).

### dATP, dCTP and dGTP measurements

Approximately 2 × 10^8 ^cells were harvested, washed with water, resuspended in 200 μL of cold 60% methanol, and extracts obtained by vigorous shacking in the presence of glass beads. Then, cell debris was pelleted at 27000 *g *for 1 min, and the supernatant was held at -20°C for 2 h. The samples were boiled for 3 min, and followed by centrifugation at 13000 *g *for 10 min at 4°C. The supernatant was taken to dryness under vacuum and resuspended in 40 μL of water. The dATP, dCTP and dGTP levels were determined by the DNA polymerase-based enzymatic assay [54]. Briefly, the incorporation by the Klenow DNA polymerase of dATP, dCTP and dGTP into specific oligonucleotides containing poly(AAAT), poly(AAAG) or poly(AAAC) sequences respectively, was determined in the presence of excess [^3^H]dTTP.

## Competing interests

The authors declare that they have no competing interests

## Authors' contributions

MSC carried out the experiments, analyzed the data and revised the manuscript. MCB carried out the experiments. J.C.I. planned, supervised, and analyzed the data and wrote the manuscript. All authors read and approved the final manuscript.
